# Multimodal Fusion of Intraoperative FLIm and Preoperative PET/CT for Patient-Level Prediction of Lymph Node Metastasis in Head and Neck Cancer

**DOI:** 10.3390/cancers18132154

**Published:** 2026-07-04

**Authors:** Lei Zhou, Nimu Yuan, Mohamed A. Hassan, Lisanne Kraft, Katjana Ehrlich, Brent W. Weyers, Vladimir Ivanovic, Osama A. A. Raslan, Dorina Gui, Marianne Abouyared, Arnaud F. Bewley, Andrew C. Birkeland, Donald Gregory Farwell, Laura Marcu, Jinyi Qi

**Affiliations:** 1 Department of Biomedical Engineering, University of California, Davis, CA 95616, USA; zhlzhou@ucdavis.edu (L.Z.);; 2 Department of Neurology, University of California, Davis, CA 95817, USA; 3 Department of Radiology, University of California, Davis, CA 95817, USA; 4 Department of Pathology and Laboratory Medicine, University of California, Davis, CA 95817, USA; 5 Department of Otolaryngology–Head and Neck Surgery, University of California, Davis, CA 95817, USA; 6 Department of Otorhinolaryngology–Head and Neck Surgery, University of Pennsylvania, Philadelphia, PA 19104, USA; 7 Department of Neurological Surgery, University of California, Davis, CA 95817, USA

**Keywords:** head and neck cancer, metastatic lymph node, PET/CT, fluorescence lifetime imaging, multimodal fusion, deep learning, patient-level prediction

## Abstract

Background: Metastatic lymph node (MLN) detection remains a major clinical challenge in head and neck cancer, as nodal involvement is strongly associated with poor prognosis and directly affects treatment planning. Previous approaches typically rely on cropped lymph node (LN) regions or tumor contours for MLN identification, requiring substantial expert annotation during preprocessing and relying solely on imaging information. As a result, small or low-contrast metastatic nodes may be missed, while benign lymph nodes may be incorrectly identified as metastatic due to overlapping imaging characteristics. To address these limitations, we propose a multimodal learning framework that integrates anatomical and metabolic features from head and neck PET/CT images with biochemical features derived from FLIm for patient-level MLN prediction, without requiring manual lymph node cropping or tumor contouring during inference. Methods: To enable robust imaging representation learning, a region-aware PET/CT network based on a merging-diverging architecture was first pretrained on the HECKTOR 2022 dataset and then fine-tuned on the institutional cohort. In parallel, FLIm point-wise measurements with clinical variables were encoded using a multilayer perceptron (MLP) and aggregated into subject-level representations. To effectively combine these modalities, two multimodal fusion strategies were evaluated at the decoder stage, including cube-based fusion and squeeze-and-excitation (SE)-based fusion. The proposed strategies were evaluated on a cohort of 53 patients. Results: Compared with the single-modality baselines, both multimodal fusion strategies achieved better patient-level MLN prediction. The PET/CT-only segmentation-driven model and FLIm-only model reached balanced accuracies of 0.815 and 0.665, with AUCs of 0.828 and 0.614, respectively. Cube-based fusion improved balanced accuracy and AUC to 0.827 and 0.850, respectively, while channel-wise SE-based fusion achieved the best overall performance, with a balanced accuracy of 0.839 and an AUC of 0.872. Conclusions: These results suggest that multimodal integration may improve patient-level MLN prediction compared with single-modality approaches. Given the limited sample size, these findings should be interpreted as hypothesis-generating and require validation in larger, independent patient cohorts.

## Simple Summary

Accurate preoperative detection of metastatic lymph nodes is critical in head and neck cancer, as it directly influences staging, treatment planning, and prognosis. However, assessment based on conventional imaging remains challenging, especially when metastatic nodes are small or lack clear morphological abnormalities. In this study, we investigate whether combining preoperative PET/CT with intraoperative fluorescence lifetime imaging (FLIm) improves patient-level prediction of metastatic lymph node status. To this end, we develop a multimodal deep learning framework that integrates imaging and biochemical information from both modalities. The results suggest that integrating PET/CT and FLIm offers a potential decision support approach for identifying patients at elevated risk of nodal metastasis. Given the limited cohort size, these findings should be considered hypothesis-generating and further validation in larger, independent patient cohorts is needed.

## 1. Introduction

Head and neck cancer (HNC) remains one of the most common cancers worldwide and continues to impose a substantial mortality and treatment burden [1,2]. Metastatic lymph node (MLN) detection is a central problem in HNC, as it is one of the strongest prognostic factors in head and neck squamous cell carcinoma (HNSCC) and is associated with markedly worse survival [3,4]. Nodal involvement is closely related to adverse oncologic outcomes, and even a single metastatic lymph node may reduce survival by up to 50 % [3,5]. Therefore, an accurate assessment of metastatic lymph node disease is essential for staging, treatment planning, and risk stratification. However, preoperative evaluation remains difficult, as the nodal status is still largely inferred from morphological imaging features such as size, shape, necrosis, cystic change, and nodal clustering. These criteria are imperfect because benign reactive nodes may be enlarged, whereas metastatic nodes may remain morphologically normal, especially in subcentimeter disease [6,7,8].

The biological heterogeneity of HNC and the subjective nature of radiological interpretation further complicate the diagnosis. As a result, up to 30 % of clinically node-negative patients may harbor occult metastases [5,9]. Missing metastatic nodal disease can delay appropriate treatment and increase the risk of recurrence, whereas overtreatment of benign lymph nodes may expose patients to treatment-related morbidity. These limitations highlight the need for reliable, noninvasive methods to improve MLN prediction in HNC.

To address this need, recent studies have explored radiomics, machine learning, and deep learning approaches for MLN detection across multiple imaging modalities. In CT-based studies, Tomita et al. developed a nodal radiomics model using contrast-enhanced CT, showing improved differentiation between benign and metastatic nodes compared with human readers in certain validation settings [10]. Xu et al. proposed a deep learning-assisted CT framework, demonstrating the feasibility of large-scale computational nodal assessment [11]. For MRI, prior work has shown that radiomics and deep learning features can support preoperative MLN prediction in oral and oropharyngeal cancers [12,13]. Ultrasound has also been used for nodal evaluation, either as an adjunct modality or in combination with radiomics and machine learning for metastasis prediction [14,15]. Among these modalities, PET/CT is particularly attractive because it combines anatomical information from CT with metabolic activity from PET. Recent systematic reviews report that PET/CT achieves a strong overall diagnostic performance compared with other imaging modalities [4,16]. Previous PET/CT studies have focused primarily on lymph node-level assessment, including hybrid radiomics and deep learning models for node classification and CNN-based approaches with attention mechanisms or uncertainty estimation [17,18,19].

Despite these advances, most MLN detection studies remain confined to routine preoperative clinical imaging, even in multimodal settings. Label-free fluorescence lifetime imaging (FLIm) captures tissue biochemical and microenvironmental characteristics through fluorescence lifetime signals, which differ from the anatomical and metabolic information provided by CT and PET. In head and neck cancer, FLIm has mainly been used for intraoperative tumor margin assessment and surgical guidance, where prior work has demonstrated its value for tissue classification and margin detection [20,21]. However, FLIm measurements of the primary tumor may also serve as a functional biomarker for the prediction of lymph node metastasis because metastatic progression in HNSCC is closely associated with metabolic reprogramming driven by epithelial–mesenchymal transition (EMT), hypoxia, and acquisition of cancer stem cell phenotypes [22]. These processes alter intracellular redox metabolism and mitochondrial function, producing measurable changes in endogenous fluorophores such as NAD(P)H and FAD. By quantifying fluorescence lifetime signatures that reflect cellular metabolic state and heterogeneity, FLIm may provide information for identifying aggressive tumor subpopulations with enhanced invasive and metastatic potential. Yuan et al. [23] showed that combining preoperative CT radiomics with intraoperative FLIm improved patient-level lymph node metastasis prediction compared with CT-only or FLIm-only models (AUC: 0.79 vs. 0.67 and 0.72). This result suggests that FLIm provides complementary biochemical information for metastatic risk assessment. However, the integration of PET/CT with FLIm for MLN prediction remains largely unexplored. This gap motivates the present study, which explores effective strategies for fusing PET/CT-derived representations with FLIm features to improve patient-level MLN prediction.

Existing MLN classification methods often rely on manually cropped lymph nodes or expert-provided tumor and nodal contours for feature extraction, requiring substantial annotation effort and limiting scalability in clinical practice. In contrast, end-to-end classification models operating on full 3D volumes avoid manual preprocessing but often lack explicit regional guidance. In this study, we develop a multimodal framework for patient-level MLN prediction that integrates intraoperative FLIm-derived biochemical information with preoperative PET/CT representations. Specifically, we adopt a segmentation-oriented, region-aware PET/CT network and investigate two FLIm-guided multimodal fusion strategies, including spatially informed cube fusion and channel-wise SE-based feature recalibration. Unlike conventional approaches that rely on explicit spatial correspondence between modalities, the proposed framework enables multimodal integration using intraoperative FLIm measurements without voxel-level registration. Patient-level MLN status is determined by converting predicted MLN regions into a binary assessment of metastatic nodal involvement. Clinically, this framework is intended to assist surgeons by integrating intraoperative FLIm measurements from the primary tumor with preoperative PET/CT to provide complementary biochemical information and enable real-time refinement of patient-level nodal risk assessment during surgical decision-making.

The main contributions of this work are as follows:

1. We propose a multimodal framework that integrates intraoperative FLIm with preoperative PET/CT for patient-level MLN prediction in head and neck cancer.
2. We investigate segmentation-driven, region-aware PET/CT feature learning for multimodal metastatic risk assessment without requiring manual lymph node cropping during inference.
3. We introduce and compare two FLIm-guided multimodal fusion strategies, including spatially informed cube fusion and SE-based channel recalibration.
4. We demonstrate that complementary biochemical information from sparse FLIm measurements can improve PET/CT-based MLN prediction despite the absence of explicit voxel-level multimodal registration.

## 2. Materials and Methods

### 2.1. Study Population

A total of 53 patients were included in this study. The cohort was collected from the Department of Otolaryngology—Head and Neck Surgery at the University of California, Davis, between 2017 and 2022 under an IRB-approved protocol. All patients underwent both preoperative PET/CT imaging and intraoperative fluorescence lifetime imaging (FLIm). The eligible patients were adults (≥18 years) with primary tumors located in the oral cavity or oropharynx. The lymph node status was determined by postoperative pathological evaluation of neck dissection specimens and served as the ground truth for patient-level prediction.

As summarized in Table 1, the cohort was predominantly male (83.0%) with a mean age of 61.9±11.1 years. The primary tumors were located mainly in the oropharynx, particularly in the palatine tonsil and tongue. Most of the patients were HPV-positive, and tobacco exposure was common in the cohort.

### 2.2. FLIm Data

Intraoperative fluorescence lifetime imaging (FLIm) data were acquired from the primary tumor region immediately prior to surgical excision using a custom FLIm system developed at the University of California, Davis (Davis, CA, USA). The system employed a 355 nm pulsed excitation laser to induce endogenous tissue autofluorescence, and point-wise measurements were collected in vivo from the exposed surgical field [24,25]. To improve signal stability, repeated measurements were averaged during acquisition.

The FLIm system recorded fluorescence responses across four spectral channels designed to capture emission bands associated with endogenous fluorophores relevant to tissue composition and metabolism, including collagen, NADH, and FAD. The spectral bands were 390 ± 20 nm, 470 ± 14 nm, 542 ± 25 nm, and 629 ± 26.5 nm. In this study, only channels 1–3 were used for the downstream analysis, while channel 4 was excluded due to its relatively low signal-to-noise ratio.

Before feature extraction, raw fluorescence waveforms were processed through background subtraction and decay analysis. Fluorescence decay characteristics were quantified using Laguerre-based deconvolution, from which lifetime-related and waveform-based descriptors were derived for each measurement point [26]. To improve robustness, low-quality measurements were removed using gain-based filtering. For each FLIm scanning point, fluorescence-derived features were extracted across the retained spectral channels, including lifetime parameters, spectral intensities, intensity ratios, Laguerre expansion coefficients, and phasor-based features, resulting in a 54-dimensional feature vector.

In addition to optical descriptors, clinical variables were incorporated to provide patient-level context, including primary tumor subsite, age, sex, HPV status, tobacco use, alcohol use, and other relevant factors. These variables were concatenated with FLIm-derived features to form a combined 64-dimensional representation for each scanning point.

### 2.3. PET/CT Data

Our preoperative PET/CT images were acquired from 17 clinical whole-body PET/CT scanner models from four vendors: GE HealthCare (Chicago, IL, USA), Siemens Healthineers (Forchheim, Germany), Philips Healthcare (Amsterdam, The Netherlands), and United Imaging Healthcare Co., Ltd. (Shanghai, China). Many scans were acquired from other institutions before the patients were referred to UC Davis. Thus, there are larger variations in the acquisition protocols and image reconstruction parameters. All PET/CT volumes were spatially aligned and resampled to isotropic 1.0 mm voxels. For each subject, a head-and-neck volume of interest was manually localized from the full-body scan. The central slices in the coronal and sagittal views were inspected, and a bounding box covering the head-and-neck region was manually defined. The bounding box was then adjusted by expansion or reduction to a fixed size of 160×160×160 voxels. Primary tumor (PT) and MLN masks were first delineated by a radiologist on conventional CT images and then transferred to the paired PET/CT images using neural correspondence field (NCF) registration method [27].

For intensity preprocessing, PET and CT volumes were normalized following the strategy described in [28]. PET images were standardized using z-score normalization. CT images were first clipped to [−1024, 1024] to suppress extreme intensity values and then linearly scaled to [−1, 1]. All preprocessing steps, including intensity normalization and bounding box definition, were performed independently of the existence of MLN in each image.

To improve representation learning despite the limited cohort size, the region-aware PET/CT network was first pretrained on the HECKTOR 2022 challenge dataset, a multi-center public benchmark for head and neck cancer PET/CT analysis [29]. During pretraining, 524 patients were included. The dataset provides ground-truth annotations for the primary gross tumor volume (GTVt) and nodal gross tumor volume (GTVn), but does not include FLIm data. It was therefore used only to pretrain the PET/CT network, while the final model was fine-tuned on our paired PET/CT and FLIm cohort. For preprocessing, PET/CT volumes were resampled to an isotropic resolution of 1 mm and cropped to a fixed size of 160×160×160 voxels, with the tumor centered within the cropped volume.

### 2.4. Overall Architecture

**Figure 1:**
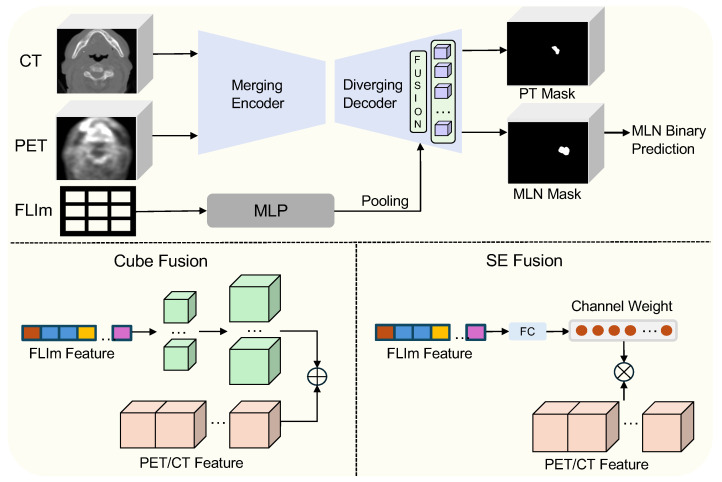
Overview of the proposed multimodal pipeline for patient-level MLN prediction through the integration of PET/CT and FLIm features. Paired three-dimensional PET/CT images are processed by a merging–diverging network to extract region-aware imaging representations, while tabular FLIm data are encoded using a multilayer perceptron (MLP). Two fusion strategies are introduced to combine PET/CT-derived imaging features with FLIm-derived biochemical information. Finally, the presence of any predicted MLN region is treated as a positive case, whereas its absence is treated as a negative case.

Figure 1 shows the overall architecture of the proposed multimodal framework for patient-level MLN prediction. The model takes paired 3D PET and CT images of the head and neck as input and processes them using a merging–diverging hybrid network [28], which extracts intra- and inter-modality features through a shared encoder. The encoded features are then passed to a diverging decoder that learns region-aware representations and produces segmentation masks for the PT and MLN.

FLIm data are represented as tabular inputs that include clinical variables and fluorescence signals. These inputs are encoded using a two-layer MLP and aggregated into subject-level features. Since each subject contains a variable number of FLIm measurement points, a max-pooling operation is applied after the MLP to obtain a fixed-length feature vector. The resulting FLIm representation is integrated with PET/CT features in the final stage of the decoder.

Two fusion strategies are considered. In cube-based fusion, the FLIm embedding is reshaped into a 3D tensor and upsampled to match the spatial resolution of the imaging features, providing a weak spatial prior that encourages the network to capture MLN-related patterns across the volume. In SE-based fusion, FLIm features are used to generate channel-wise weights through a fully connected layer, which are then applied to reweight PET/CT feature maps and emphasize features relevant to MLN detection.

Finally, the predicted MLN segmentation mask is converted into a patient-level binary outcome: the presence of any predicted MLN region is treated as a positive case, whereas its absence is treated as a negative case. In this way, the model produces a binary classification of whether the patient has metastatic lymph nodes.

### 2.5. PET/CT Feature Extraction

The PET and CT feature extraction backbone is adapted from XSurv [28], a dual-branch merging–diverging architecture originally designed for joint tumor segmentation and survival prediction. In this work, we retain the segmentation-driven design while removing the survival prediction head, resulting in a segmentation-only variant.

In the encoder, PET and CT features are first processed independently and then fused through Hybrid Parallel Cross-Attention (HPCA) blocks, which combine convolutional operations for local intra-modality feature extraction with cross-attention mechanisms for global inter-modality interaction. This design integrates anatomical and metabolic information while preserving modality-specific characteristics.

After the merging stage, a diverging decoder is used to learn region-specific representations. The Region Attention Gate (RAG) blocks generate mutually exclusive attention maps, allowing the network to separate shared features into PT and MLN components.

This merging–diverging structure produces cross-modality, region-aware feature representations from PET/CT data, which serve as the basis for subsequent multimodal fusion and patient-level prediction.

To train this region-aware segmentation backbone, we use a hybrid loss defined as the sum of Dice loss [30] and focal loss [31]. Dice loss encourages spatial overlap between the predicted masks and the ground-truth PT and MLN annotations:

(1) $$L_{\mathrm{Dice}}=1-\frac{2\sum_{i}p_{i}g_{i}+\epsilon }{\sum_{i}p_{i}+\sum_{i}g_{i}+\epsilon },$$

where $p_{i}$ and $g_{i}$ denote the predicted probability and the ground-truth label in voxel *i*, respectively, and ϵ is a small constant for numerical stability. Focal loss is used to emphasize hard voxels and address foreground–background imbalance:

(2) $$L_{\mathrm{Focal}}=-\frac{1}{N}\sum_{i}\alpha {\left(1-q_{i}\right)}^{\gamma }\log\left(q_{i}\right),$$

where *N* is the number of voxels, $q_{i}$ denotes the predicted probability assigned to the true class at voxel *i*, clipped to the interval [ϵ,1−ϵ] with $\epsilon =10^{-5}$ for numerical stability, α=0.25 is the class-balancing factor, and γ=2 is the focusing parameter. The segmentation loss was defined as the equally weighted sum of the Dice loss and the focal loss.

### 2.6. FLIm Feature Extraction

The input FLIm data are represented by 64-dimensional feature vectors that include fluorescence lifetime parameters and relevant clinical variables. Each feature vector is encoded into a latent representation using a multilayer perceptron (MLP):

(3) $$z_{i}=\phi \left(f_{i}\right),\;z_{i}\in R^{128},$$

where $f_{i}\in R^{64}$ denotes the feature vector of the *i*-th scanning point, and ϕ(·) represents the MLP encoder. Since the number of FLIm scanning points varies across subjects, the data cannot be directly represented as a fixed-length subject-level vector. To address this, the encoded features are then aggregated across all scanning points for each subject using max pooling. The resulting representation captures the most informative biochemical signals and is used for subsequent multimodal feature fusion.

### 2.7. Feature Fusion

To integrate fluorescence lifetime imaging (FLIm) signals with PET/CT features, we introduce two FLIm-guided fusion mechanisms applied at the final stage of the decoder. Let $X\in R^{C\times H\times W\times D}$ denote the PET/CT feature map from the decoder, and $z\in R^{128}$ denote the pooled FLIm feature vector.

#### 2.7.1. Cube-Based Fusion

To incorporate spatial structure, we adopt a cube-based fusion strategy that maps FLIm features into a low-resolution volumetric representation. The vector *z* is reshaped into a 3D tensor:

(4) $$z_{\mathrm{cube}}\in R^{C^{\prime }\times h\times w\times d},$$

where $C^{\prime }\cdot h\cdot w\cdot d=128$. The tensor is then upsampled to match the spatial resolution of the decoder feature map:

(5) $$\tilde{z}=\mathrm{Upsample}\left(z_{\mathrm{cube}}\right)\in R^{C^{\prime }\times H\times W\times D}.$$

The upsampled representation is concatenated with PET/CT feature map *X* along the channel dimension:

(6) $$X^{\prime }=\mathrm{Concat}\left(X,\tilde{z}\right),$$

and processed by subsequent convolutional layers to produce the final segmentation output.

This formulation introduces a spatially informed latent representation derived from FLIm signals. Although FLIm measurements are sparse, the learned cube representation allows the network to project biochemical information into a spatially structured latent space. This provides additional spatial guidance for the decoder, which is useful for identifying metastatic lymph node regions.

#### 2.7.2. SE-Based Fusion

**Figure 2:**
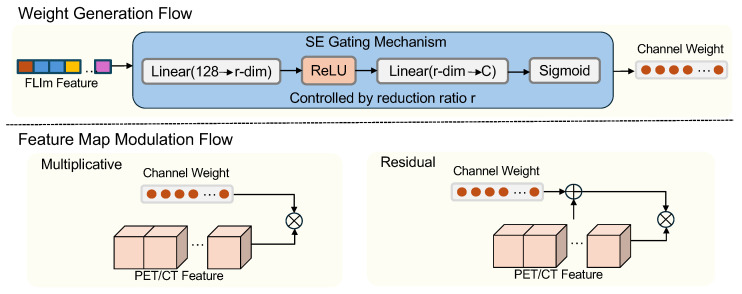
Overview of the proposed SE-based fusion strategy for combining FLIm and PET/CT features. A pooled subject-level FLIm representation is first passed through a lightweight SE-style gating network to generate channel-wise weights for the PET/CT decoder feature map. The hidden dimension of the gating module is controlled by the reduction ratio *r*. Two modulation forms are considered: multiplicative modulation, which directly rescales the decoder channels, and residual modulation, which recalibrates the channels while preserving the original PET/CT feature responses through an identity-preserving form.

We also consider a channel-wise modulation strategy inspired by the squeeze-and-excitation (SE) mechanism [32]. In contrast to the standard SE formulation, where channel weights are derived from image features, the modulation weights are computed from FLIm features. As shown in Figure 2, the pooled FLIm feature vector $z\in R^{128}$ is first fed into a lightweight gating network that produces channel-wise weights for the PET/CT decoder feature map. Specifically, *z* is passed through two fully connected layers with a ReLU activation between them, followed by a sigmoid function:

(7) $$s=\sigma \left(\psi \left(z\right)\right),\;s\in R^{C},$$

where ψ(·) denotes the learnable projection from the 128-dimensional FLIm embedding to the channel space of the target decoder feature map, σ(·) is the sigmoid activation, and *C* is the number of feature channels.

The hidden dimension of the gating network is controlled by a reduction ratio *r*, as illustrated in the upper part of Figure 2. The first linear layer reduces the 128-dimensional FLIm feature to a bottleneck representation, and the second maps this representation to *C* channel weights. The value of *r* therefore controls the capacity of the gating module: a smaller *r* gives a larger bottleneck dimension and a more flexible modulation function, while a larger *r* introduces stronger compression and lowers model complexity. In this study, we evaluated multiple values of *r* to examine how the bottleneck size affects FLIm-guided channel recalibration.

Given a PET/CT decoder feature map $X\in R^{C\times H\times W\times D}$, the generated weight vector *s* is broadcast across the spatial dimensions and used to modulate the feature channels. As shown in the lower part of Figure 2, we considered two modulation strategies. In multiplicative fusion, the channel weights directly rescale the PET/CT feature channels:

(8) $${\tilde{X}}_{\mathrm{mul}}=s⊙X,$$

where ⊙ denotes channel-wise multiplication. This allows FLIm to directly adjust the relative contribution of different PET/CT channels. In residual fusion, the modulation is applied in an identity-preserving form:

(9) $${\tilde{X}}_{\mathrm{res}}=\left(1+s\right)⊙X.$$

This formulation preserves the original feature responses while incorporating FLIm-guided adjustments, leading to more stable modulation.

These formulations allow FLIm features to modulate channel importance globally, effectively conditioning the decoder on biochemical information and enabling adaptive feature recalibration.

### 2.8. Implementation Details

The PET/CT backbone was first pretrained on the HECKTOR 2022 dataset and subsequently fine-tuned using our institutional cohort. The HECKTOR 2022 and UC Davis cohorts are independent with no patient-level overlap. During pretraining, 415 patients from five centers (CHUP, MDA, CHUS, HMR, and HGJ) in the HECKTOR 2022 dataset were used for training, while 109 patients from two centers (CHUM and CHUV) were used for validation. Among the 415 training cases, 48 were node-negative and 367 were node-positive. To mitigate class imbalance during pretraining, node-negative cases were oversampled by a factor of nine.

Our institutional cohort consisted of 53 subjects from UC Davis. To reduce the effect of data partition bias and training stochasticity, we used 11 random seeds for dataset splitting and model initialization, with 13 subjects assigned to the test set in each split. Dataset splits were performed at the patient level to ensure that no data from the same subject appeared in both the training and the test set. Because of the limited cohort size, performance was averaged across repeated random splits rather than reported from a single train–test partition. For each split, the held-out test set was used only for final evaluation. Hyperparameter tuning was not performed using test data and the model architecture and training settings were fixed across repeated splits. Final performance was reported as the average across all runs. To assess whether performance differences between models were statistically significant, we conducted paired Wilcoxon signed-rank tests on model performance across the 11 matched random splits. A significance level of *p* < 0.05 was used.

For both pretraining and fine-tuning, the same augmentation strategy was applied to increase data diversity, following the implementation in [28]. PET/CT images and their corresponding PT and MLN masks were augmented jointly to maintain spatial alignment across modalities and annotations. The augmentation pipeline included random affine transformations, such as translation, scaling, shearing, and rotation, followed by random cropping to 112×112×112. After augmentation, the PT and MLN masks were binarized using a threshold of 0.5.

The model was implemented in PyTorch and trained on a single NVIDIA A100 GPU with 80 GB of memory (NVIDIA Corporation, Santa Clara, CA, USA). Optimization was performed using the Adam optimizer with a learning rate schedule decayed from $1\times 10^{-3}$ to $1\times 10^{-6}$. All experiments were conducted in a conda environment on a Linux computer using Python 3.8.0, PyTorch 2.0.0, CUDA 11.8, and cuDNN 8.7.0.

## 3. Results

### 3.1. Overall Result

Table 2 summarizes the patient-level MLN classification performance of all methods on the test set. Sensitivity, specificity, and balanced accuracy are reported as mean ± standard deviation over 11 matched random runs, while AUC was computed by pooling all subject-level predictions across all runs. Among the single-modality baselines, the FLIm-only model achieved the lowest balanced accuracy (0.665±0.086) and AUC (0.614), suggesting that FLIm-derived biochemical features from PT alone provide limited discriminative power without imaging context. For the PET/CT-only baselines, PET/CT-cla used imaging features extracted from the pretrained encoder of the region-aware PET/CT network as input to an MLP classifier, achieving a balanced accuracy of 0.701±0.133 and an AUC of 0.657. In contrast, PET/CT-seg used the full encoder–decoder network to generate region-aware MLN prediction maps, resulting in improved performance with a balanced accuracy of 0.815±0.162 and an AUC of 0.828. This comparison indicates that segmentation-driven region-aware representations are more effective for patient-level MLN prediction than encoder-level features alone.

Both multimodal fusion methods further improved performance over the single-modality baselines. Cube Fusion achieved a balanced accuracy of 0.827±0.093 and an AUC of 0.850, while SE Fusion achieved the best overall performance with the highest sensitivity (0.899±0.090), balanced accuracy (0.839±0.126), and AUC (0.872). Although Cube Fusion and SE Fusion had the same mean specificity (0.780), Cube Fusion showed a smaller standard deviation in specificity (0.164 vs. 0.221), suggesting slightly more stable identification of negative cases. Overall, these results indicate that incorporating FLIm information into region-aware PET/CT representations improves patient-level MLN classification, with SE Fusion providing the strongest discriminative performance.

To further assess the reliability of patient-level predictions for potential clinical decision support applications, we evaluated both discriminative performance and probability calibration using subject-level scores derived from the cc_top50 strategy. In this strategy, connected components were first identified from the predicted MLN probability map. When connected components were detected, the component with the highest probability response was selected, and the mean probability of its top 50 voxels was used as the subject-level score. If no connected component was detected, the mean probability of the global top 50 voxels was used instead.

**Figure 3:**
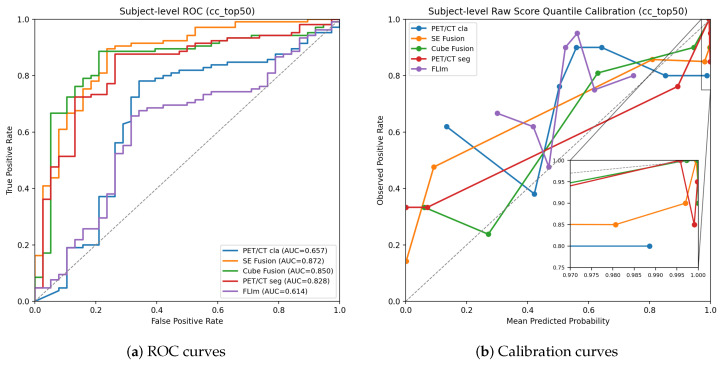
Comparison of patient-level discrimination and calibration performance across different methods. (**a**) ROC curves based on subject-level scores computed using the cc_top50 strategy. SE Fusion achieves the highest discriminative performance (AUC = 0.872), followed by Cube Fusion, indicating that fusing PET/CT with FLIm information improves the model’s ability to distinguish patients with MLN. (**b**) Calibration curves using quantile-based binning on raw prediction scores. The fusion methods show overall favorable calibration, with Cube Fusion and SE Fusion generally close to the ideal diagonal. PET/CT-seg also shows reasonable calibration, although deviations remain in high-probability regions.

Figure 3 compares patient-level ROC and calibration performance using these scores. ROC curves were computed from pooled subject-level predictions across the 11 runs, while calibration curves were generated using quantile-based binning with 7 bins. SE Fusion achieved the highest AUC, followed by Cube Fusion, and both fusion methods showed better calibration than the unimodal baselines.

The quantitative calibration results are summarized in Table 3. Both fusion methods improved calibration compared with unimodal baselines. Cube Fusion achieved the lowest Brier score (0.139±0.023) and ECE (0.103±0.026), followed closely by SE Fusion (0.140±0.026 and 0.129±0.028). By contrast, PET/CT-cla, PET/CT-seg, and FLIm showed higher Brier scores and ECE values.

**Figure 4:**
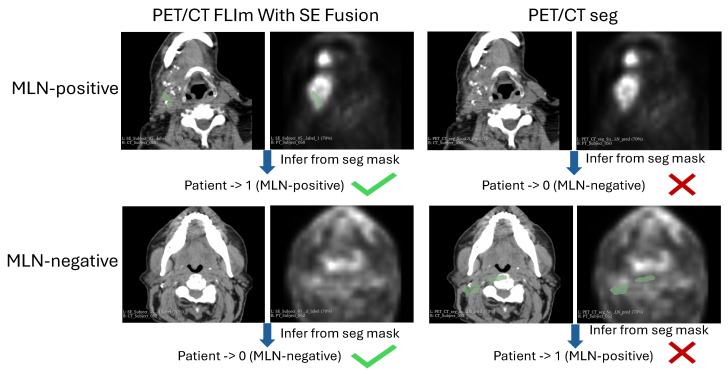
Representative qualitative comparison between the proposed SE-based fusion model and the PET/CT-seg model for patient-level MLN prediction. The top row shows an MLN-positive case, and the bottom row shows an MLN-negative case. Green overlays indicate predicted MLN regions, and patient-level predictions inferred from the segmentation masks are shown below each example. Green check marks indicate correct patient-level predictions, whereas red crosses indicate incorrect patient-level predictions. SE Fusion produced the correct patient-level prediction in both examples, whereas PET/CT-seg resulted in incorrect predictions.

Figure 4 presents representative qualitative comparisons between SE Fusion and PET/CT-seg. In the MLN-positive example, SE Fusion correctly identified the patient as positive, whereas PET/CT-seg missed the lesion and classified the patient as negative. In the MLN-negative example, PET/CT-seg generated false-positive MLN responses and incorrectly classified the patient as positive, while SE Fusion suppressed these spurious predictions and correctly classified the patient as negative. This visual comparison is consistent with the quantitative results in Table 2, supporting the benefit of multimodal fusion over PET/CT-only prediction.

### 3.2. Additional Statistical and Clinical Analyses

To assess the incremental value of multimodal fusion, the SE Fusion model was compared with the PET/CT-seg baseline. AUC confidence intervals and the paired AUC difference were estimated using 10,000 stratified bootstrap samples, and paired ROC curves were compared using DeLong’s test. As shown in Table 4, SE Fusion achieved an AUC of 0.872 (95% CI: 0.799–0.933), compared with 0.828 (95% CI: 0.746–0.898) for PET/CT-seg. The resulting ΔAUC was 0.045 (95% CI: −0.001 to 0.091). The improvement did not reach the prespecified significance threshold (DeLong p=0.055), indicating a trend toward improved performance that should be validated in larger datasets.

**Figure 5:**
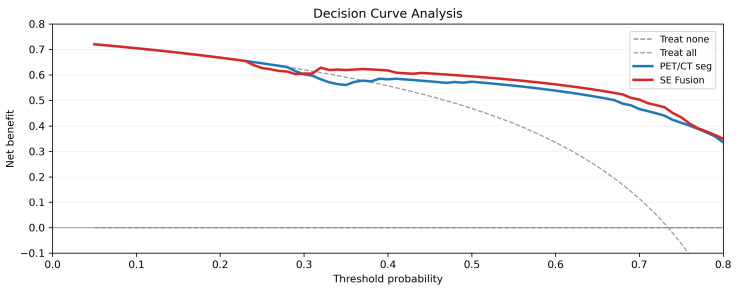
Decision curve analysis comparing SE Fusion with the PET/CT-seg baseline for patient-level MLN prediction. Treat-all and treat-none strategies are shown as reference curves.

We further assessed clinical utility using continuous net reclassification improvement (NRI) and decision curve analysis (DCA). SE Fusion yielded a total NRI of 1.203 (95% CI: 0.891–1.494), with an event NRI of 0.676 and a nonevent NRI of 0.526. The positive event and nonevent components indicate that predicted risks shifted in the expected direction for both outcome groups. The DCA in Figure 5 demonstrated that SE Fusion consistently achieved higher net benefit than PET/CT-seg, particularly between threshold probabilities of approximately 0.3 and 0.7, indicating improved clinical utility for risk-guided decision-making.

**Figure 6:**
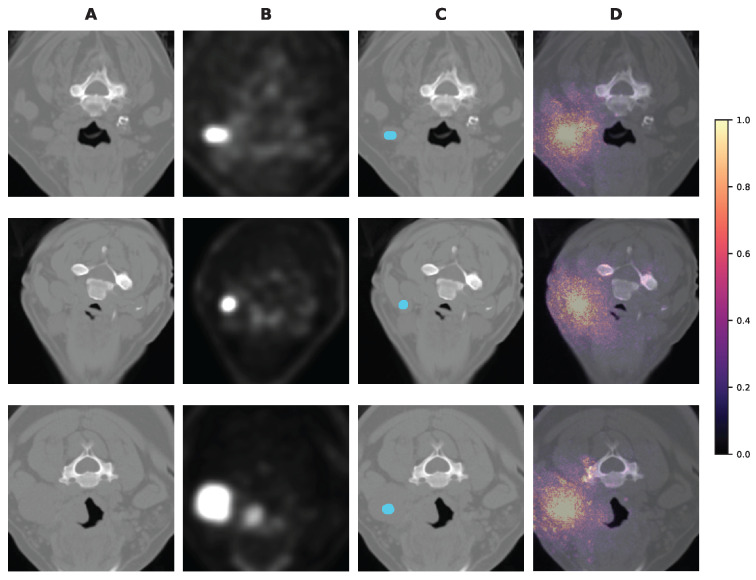
Representative integrated gradients (IG) visualizations generated by the SE fusion model for MLN prediction. Rows show representative axial slices from three cases. (**A**) CT images, (**B**) corresponding PET images, (**C**) CT images with the top-50 MLN output voxel locations shown in blue, and (**D**) normalized IG attribution maps overlaid on CT. IG was computed with respect to the input PET/CT voxels using the mean of the top-50 pre-sigmoid MLN logits from the original prediction as the scalar target. The color scale indicates visualization-normalized IG attribution from 0 to 1. These visualizations qualitatively highlight input regions related to the model’s selected MLN prediction response.

To further assess model interpretability, we generated integrated gradients (IG) attribution maps to visualize the PET/CT regions contributing to MLN prediction. For each case, the mean of the top-50 pre-sigmoid MLN logits from the original prediction was used as the scalar target. The resulting IG maps were overlaid on CT for anatomical reference. As shown in Figure 6, the maps highlighted localized regions with the greatest contribution to model prediction, providing a qualitative view of the image regions influencing model output. Additional SE-weight analyses are provided in Supplementary Figures S1 and S2.

We further performed an exploratory analysis of SE Fusion performance across available clinical subgroups, including HPV status, primary tumor site, and tobacco use. The results are shown in Table 5. Because several subgroups contained small sample sizes and limited numbers of MLN-negative cases, performance metrics were calculated from pooled predictions across random seeds rather than from seed-level averages; consequently, standard deviations are not reported. Overall, model performance was generally consistent across most subgroups, with AUCs ranging from 0.812 to 0.907 among the larger strata. However, these subgroup analyses should be interpreted with caution, as the resulting estimates may be unstable. For example, the lower AUC observed in HPV-negative patients, as well as the near-perfect AUCs observed in the base-of-tongue and current-smoker subgroups, were likely influenced by the very small number of MLN-negative cases in these strata. No formal statistical comparisons between subgroups were performed.

### 3.3. Ablation Study

Table 6 presents the ablation results of cube-based fusion variants for patient-level MLN classification. In this setting, the 128-dimensional FLIm feature vector produced by the MLP was reshaped into different 3D cube configurations, followed by volumetric upsampling and fusion with the decoder feature map of the region-aware PET/CT network. Here, *s* denotes the spatial size of the reshaped cube. Given that the final MLN-related PET/CT feature map had 8 channels, two cube configurations were evaluated: Cube s2, corresponding to a 16×2×2×2 representation, and Cube s4, corresponding to a 2×4×4×4 representation. Thus, the same 128-dimensional FLIm feature vector was arranged into different spatial layouts before upsampling.

The two cube configurations showed comparable performance. Cube s2 achieved slightly higher sensitivity (0.878±0.115 vs. 0.873±0.089), whereas Cube s4 yielded higher specificity (0.780±0.164 vs. 0.773±0.221), a marginally higher balanced accuracy (0.827±0.093 vs. 0.826±0.089), and a slightly higher AUC (0.850 vs. 0.842). Cube s4 also showed lower variability in sensitivity and specificity. These results suggest that, although the influence of the spatial reshaping strategy was limited, the Cube s4 configuration provided a slightly more stable trade-off between positive and negative case recognition.

Table 7 presents the ablation results of SE-based fusion variants for patient-level MLN classification, and Table 8 summarizes the corresponding gating structures for different reduction ratios *r*. In this design, the pooled 128-dimensional FLIm feature vector was used to generate channel-wise modulation weights for the final MLN-related decoder feature map. Two modulation strategies were evaluated: direct multiplicative modulation (mu) and residual modulation (residual), with reduction ratios r∈0,4,16. As shown in Table 8, *r* determines the hidden dimensionality of the gating branch. Briefly, r=0 denotes a single linear mapping, whereas r=4 and r=16 introduce nonlinear bottleneck gating with hidden dimensions of 32 and 8, respectively.

Among all configurations, SE (mul, r=16) achieved the best overall performance, with a sensitivity of 0.899±0.090, a specificity of 0.780±0.221, a balanced accuracy of 0.839±0.126, and an AUC of 0.872. Within the multiplicative setting, r=16 outperformed both r=0 and r=4, indicating that a compact nonlinear gating structure is more effective for FLIm-guided channel modulation. Although SE (mul, r=16) introduces an additional nonlinear layer, its parameter count remains comparable to that of r=0 (1104 vs. 1032), suggesting that the improvement is not simply due to increased model capacity. By contrast, the r=4 configuration has the largest parameter count (4392) but does not yield better performance, further indicating that a larger gating branch alone does not improve fusion. Within the residual setting, SE (residual, r=0) achieved the best balanced accuracy (0.836±0.090), the highest AUC (0.851), and the highest specificity (0.795±0.141), while stronger bottleneck compression with r=4 or r=16 did not lead to further gains. Overall, the effect of the reduction ratio depends on the modulation form: multiplicative modulation benefits from compact nonlinear gating, whereas residual modulation performs best with a simple linear mapping.

## 4. Discussion

The results in Table 2, Table 6 and Table 7 provide a unified view of model performance for patient-level MLN prediction. To the best of our knowledge, this is the first study to investigate integration of intraoperative FLIm with preoperative PET/CT for patient-level MLN prediction in head and neck cancer. Across all experiments, multimodal models consistently outperformed both PET/CT-only and FLIm-only baselines, demonstrating the advantage of integrating heterogeneous information sources. At the same time, the integration strategy plays a key role. The differences between PET/CT-cla and PET/CT-seg, as well as between Cube Fusion and SE Fusion, suggest that performance depends not only on the inclusion of multiple modalities, but also on whether the fusion design aligns with the characteristics of both modalities.

Table 2 further highlights the impact of architectural design and multimodal fusion on patient-level MLN prediction. The performance gap between PET/CT-cla and PET/CT-seg shows that predictive performance depends strongly on how PET/CT information is used. Although both approaches rely on PET/CT-derived features, directly applying a classifier to encoder features leads to substantially lower performance than the segmentation-driven region-aware network. This suggests that MLN prediction benefits from region-aware feature learning that emphasizes lesion-relevant anatomical and pathological information, rather than relying solely on global representations. The region-aware decoder likely enables the network to focus on lesion-related cues that are more closely associated with the nodal status.

Our PET/CT-only performance for patient-level prediction is comparable to those reported in existing studies [16]. It is important to distinguish our task from prior PET/CT-based lymph node-level studies [17,18,19], which reported higher AUCs. These approaches were designed for candidate LN classification and required manually cropped PET/CT patches containing the target lymph node during inference. In contrast, our framework takes a head-and-neck PET/CT volume as input without manually specified LN locations during inference and performs end-to-end patient-level MLN prediction. This setting requires the model to learn MLN-related regions from the image itself, making the task more challenging and not directly comparable to pre-localized lymph node ROI classification.

FLIm-only results indicate that FLIm measurements carry useful biochemical information, but are insufficient for a robust prediction of lymph node metastasis on its own. This is expected, as FLIm in this study is acquired from the primary tumor region as sparse point-based measurements and does not directly assess lymph nodes. Still, FLIm captures biochemical tissue characteristics of the primary tumor related to cellular metabolism and microenvironment. Both fusion strategies consistently outperform the PET/CT-only segmentation baseline, supporting the view that FLIm provides additional information beyond conventional imaging. This improvement is clinically meaningful, as metastatic lymph node assessment depends on multiple factors, including morphologic and metabolic patterns captured by CT and PET, as well as biochemical characteristics that may be reflected by FLIm. Therefore, the results suggest that the integration of intraoperative optical measurements with preoperative PET/CT can improve patient-level risk stratification.

Among the two fusion strategies, SE Fusion achieved the best overall performance, particularly in sensitivity, balanced accuracy, and AUC. This suggests that, in the present setting, FLIm information is more effectively integrated through channel-wise modulation than through an explicit spatial prior. This observation is consistent with the nature of FLIm acquisition in this study. Since FLIm is not available as a dense 3D image but as pooled subject-level information derived from sparse sampling points, using it to recalibrate PET/CT feature channels is more consistent with its data characteristics than imposing a spatial cube representation. By contrast, Cube Fusion introduces a spatial prior that is less aligned with the inherently sparse and nonuniform nature of FLIm measurements. At the same time, the lower standard deviation of specificity and better calibration scores observed with Cube Fusion suggest slightly more stable performance, indicating a trade-off between peak accuracy and robustness.

Although SE Fusion achieved the highest overall AUC, its incremental improvement over PET/CT-seg was modest (ΔAUC = 0.045) and did not reach statistical significance by the paired DeLong test, while the bootstrap confidence interval for the AUC difference slightly crossed zero. Nevertheless, the NRI and DCA results provide complementary evidence regarding the potential clinical value of multimodal fusion. Positive event and nonevent NRI values indicate that SE Fusion frequently shifted predicted risk upward for patients with MLN and downward for patients without MLN. DCA further showed higher net benefit than PET/CT-seg across a broad range of threshold probabilities. Together, these findings suggest that multimodal fusion may improve risk stratification, particularly for patients with intermediate or equivocal PET/CT-based risk, for whom additional risk information could help inform neck-management decisions or targeted nodal assessment. The model interpretation analyses provide spatial and feature-level insight. The integrated gradients visualizations complement the quantitative evaluation by providing an input-level view of PET/CT image regions contributing to MLN predictions. The attribution patterns were spatially localized rather than broadly distributed across the image background, suggesting that the model prediction was associated with focused PET/CT features.

The ablation results in Table 6 and Table 7 provide further insight into how FLIm information is integrated with PET/CT features for patient-level MLN prediction. Variations within the cube-based fusion design led to only minor performance differences, whereas the SE-based variants showed clearer separation and better overall results. This suggests that the key factor is not how FLIm features are reshaped, but how they are incorporated into the PET/CT representation. The SE-based variants suggest that channel-wise modulation is better suited to the nature of FLIm in this setting. Rather than assuming spatial correspondence, SE Fusion uses FLIm as a global conditioning signal to recalibrate PET/CT feature channels. This design is consistent with the fact that the FLIm representation is not spatially registered to PET/CT. Among all tested variants, SE (mul, r=16) achieved the best overall performance, while SE (residual, r=0) achieved the highest specificity within the SE group. These results indicate that both the modulation form and the gating design affect fusion effectiveness. One possible explanation is that multiplicative modulation depends more directly on the gating signal, since the learned weights scale the PET/CT feature channels themselves. Under this formulation, a compact nonlinear gating branch may provide enough flexibility to learn discriminative FLIm-guided channel weights while keeping the increase in parameters limited. In contrast, residual modulation preserves the original PET/CT features through the identity path, making the gating branch an auxiliary adjustment rather than the main controller of feature strength.

From a clinical perspective, the proposed PET/CT–FLIm framework offers a practical strategy for multimodal nodal risk stratification in head and neck cancer. PET/CT provides preoperative structural and functional information, whereas FLIm provides optical-biochemical assessment of the primary tumor or tumor-adjacent mucosa. By combining these complementary sources, the framework allows biochemical signatures from the primary tumor to contribute to metastatic risk assessment, adding information beyond conventional imaging alone. This may be especially relevant when neck management is planned after resection of an oral cavity primary tumor, while pathologic staging, depth of invasion, and other risk features are still pending. It may also be relevant in oropharyngeal cancer, where operative sequencing can depend on robotic access limitations and surgeon preference. In these settings, PET/CT–FLIm fusion may help guide risk-adapted neck management when FLIm acquisition and model inference occur before the relevant neck-management decision has been finalized, by identifying patients for whom unnecessary neck dissection could potentially be avoided, while also supporting neck dissection or targeted nodal sampling when PET/CT findings are equivocal. This timing caveat is important because FLIm was acquired intraoperatively in the present study. Therefore, the full PET/CT–FLIm output should be interpreted as perioperative decision support rather than as a fully preoperative treatment-planning tool, and it would only be expected to inform surgical or perioperative decisions that remain open after FLIm data become available. This approach is more clinically actionable if oral cavity lesions, including oral tongue tumors, and endoscopically accessible oropharyngeal lesions can be evaluated with FLIm in the outpatient or preoperative setting. Under such a future workflow, FLIm-derived biochemical information could be available during definitive surgical planning, further supporting the use of PET/CT–FLIm fusion for personalized, risk-adapted management of the neck.

Practical implementation of the PET/CT–FLIm framework would require consideration of workflow integration, equipment availability, operator training, and cost-effectiveness. In this study, FLIm acquisition was performed using a handheld device and typically required approximately one minute per scan. Model inference required approximately 1 s on a single NVIDIA A100 80 GB GPU, suggesting that model inference itself is unlikely to be a major bottleneck and could support near-real-time intraoperative prediction once PET/CT and FLIm data are available. However, a fully integrated operating-room workflow was not prospectively evaluated, and the hardware requirements for clinical deployment remain to be determined. Future prospective studies should assess deployment requirements, clinical workflow integration, and cost-effectiveness.

The contribution of this work is not limited to the performance gain obtained by adding FLIm to PET/CT. It also shows that heterogeneous clinical data streams can be integrated for metastatic risk assessment. The SE-based fusion model provides one example of how preoperative anatomic-functional imaging and intraoperative optical-biochemical information can be combined within a single decision support framework. The same multimodal framework can also be applied to fluorescence-guided surgery, where preoperative imaging and intraoperative measurements can be integrated to improve intraoperative assessment of tumor metastases [33].

This study has several limitations. First, the cohort size is relatively small (n = 53), with a limited number of MLN-negative cases, which may restrict generalizability, contribute to variability across dataset splits, and reduce statistical power to detect modest differences between competing models. Although repeated random splitting and pretraining on the HECKTOR dataset were used to improve robustness, the possibility of overfitting remains due to the limited sample size. Second, the FLIm data were acquired at a single institution using research instrumentation developed at our institution and not yet widely available at other centers, which currently limits external validation. Although the cohort included cases from multiple surgeons and FLIm measurements were collected using two imaging systems (V4 and FLImBrush) under a unified acquisition protocol, these factors provide only within-institution variability and do not establish reproducibility across institutions. Future multi-center studies should standardize and harmonize FLIm acquisition protocols, including device calibration, tumor sampling strategy, working distance, and operator training. In contrast, many preoperative PET/CT scans were acquired from outside institutions using 17 scanner models from 4 vendors and a range of reconstruction protocols, introducing technical heterogeneity that may have affected model performance but may also better reflect real-world imaging variability. Future multi-center studies will be important for assessing generalizability and broader clinical applicability. Third, FLIm measurements are not spatially registered to PET/CT, which limits the ability to perform explicit spatially aligned multimodal fusion and motivates the development of more spatially informed multimodal integration strategies. This constraint may have reduced the ability to fully leverage complementary spatial information and motivates the development of more spatially informed multimodal integration strategies. Fourth, the reference standard was based on pathologic assessment in patients who underwent neck dissection. The study did not include a separate cohort of patients managed without neck dissection and followed longitudinally to assess subsequent regional recurrence. Therefore, occult nodal disease in non-dissected patients could not be evaluated, and negative predictions should be interpreted in the context of a surgically treated cohort. Finally, this retrospective study should be viewed as a feasibility and hypothesis-generating analysis rather than evidence for immediate clinical implementation. Prospective validation will be required to determine whether this workflow is feasible in real-world surgical settings, whether model predictions remain accurate before final pathology is known, and whether the model output can meaningfully affect surgical or perioperative decision-making. Future work should focus on prospective multi-center validation and workflow integration to determine the generalizability, clinical utility, and practical feasibility of PET/CT–FLIm fusion for MLN prediction.

## 5. Conclusions

In this study, we developed a multimodal framework for patient-level prediction of metastatic lymph node status in head and neck cancer by integrating PET/CT and FLIm information. The results show that combining multiple sources of information leads to better overall performance than single-modality models based on PET/CT or FLIm alone, highlighting the benefit of multimodal fusion for MLN prediction. Among the proposed methods, SE-based fusion achieves the best overall performance, suggesting that channel-wise feature modulation is more effective than spatial prior injection in the current setting. In general, these results demonstrate the potential value of multimodal fusion for MLN prediction and provide a basis for future work on integrating heterogeneous clinical and imaging information in head and neck cancer. However, considering the limited sample size and single-center nature of this study, the present findings should be interpreted as hypothesis-generating. Confirmation in larger, independent, preferably prospective multi-center cohorts will be necessary before broader clinical translation.

## Figures and Tables

**Table 1:** Clinical and demographic characteristics of the study cohort (n = 53).

Variable	Value
*Patient demographics*	
Age (years)	61.9±11.1
Male	44 (83.0%)
Female	9 (17.0%)
*Primary tumor location*	
Palatine tonsil	23 (43.4%)
Oral tongue	12 (22.6%)
Base of tongue	11 (20.8%)
Other sites	7 (13.2%)
*Metastatic lymph node status*	
MLN-Negative	15 (28.3%)
MLN-Positive	38 (71.7%)
*HPV status*	
Positive	32 (60.4%)
Negative	11 (20.8%)
Unknown	10 (18.9%)
*Tobacco use*	
Non-smoker	24 (45.3%)
Former smoker	23 (43.4%)
Current smoker	6 (11.3%)

**Table 2:** Comparison of different methods for patient-level MLN classification on the test set. PET/CT-cla and PET/CT-seg are PET/CT-only baselines, where PET/CT-cla uses features extracted from the frozen PET/CT encoder for direct classification and PET/CT-seg uses the region-aware PET/CT encoder–decoder model. FLIm denotes classification using FLIm tabular features only. Cube Fusion and SE Fusion represent the two proposed multimodal fusion strategies. Sensitivity, specificity, and balanced accuracy are reported as mean ± standard deviation over 11 random runs, while AUC was computed from pooled subject-level predictions across all runs. Statistical significance was assessed using the paired Wilcoxon signed-rank test.

Method	Sensitivity	Specificity	Balanced Acc	AUC
PET/CT-cla *	0.781±0.160	0.621±0.240	0.701±0.133	0.657
PET/CT-seg *	0.872±0.110	0.758±0.308	0.815±0.162	0.828
FLIm *	0.655±0.173	0.674±0.259	0.665±0.086	0.614
Cube Fusion	0.873±0.089	0.780±0.164	0.827±0.093	0.850
SE Fusion	0.899±0.090	0.780±0.221	0.839±0.126	0.872

**Note:** Bold values indicate the best performance for each metric. * indicates that SE Fusion significantly outperformed the corresponding baseline method (*p* < 0.05, paired Wilcoxon signed-rank test).

**Table 3:** Calibration performance of different methods at the patient level. Patient-level probabilities were computed using the cc_top50 strategy. Brier score and Expected Calibration Error (ECE) are reported as mean ± standard deviation over 1000 bootstrap resamples with replacement. ECE was calculated using quantile-based binning with 7 bins. Lower values indicate better calibration. Statistical significance was assessed using the paired Wilcoxon signed-rank test.

Method	Brier	ECE
PET/CT-cla *	0.249±0.022	0.252±0.032
PET/CT-seg *	0.163±0.030	0.152±0.031
FLIm *	0.243±0.011	0.236±0.033
Cube Fusion	0.139±0.023	0.103±0.026
SE Fusion *	0.140±0.026	0.129±0.028

**Note:** Bold values indicate the best performance for each metric. * indicates that the corresponding method performed significantly worse than Cube Fusion (p<0.05, paired Wilcoxon signed-rank test).

**Table 4:** Discrimination performance of the evaluated models for patient-level MLN prediction. AUC values are presented with 95% confidence intervals estimated using 10,000 stratified bootstrap resamples.

Model	AUC	95% CI
PET/CT-cla	0.657	0.542–0.767
PET/CT-seg	0.828	0.746–0.898
FLIm	0.614	0.508–0.717
Cube Fusion	0.850	0.770–0.919
SE Fusion	0.872	0.799–0.933

**Table 5:** Exploratory subgroup performance analysis of the SE Fusion model stratified by available clinical variables. Metrics were computed from pooled prediction results across seeds because several subgroups contained a limited number of patients; consequently, standard deviations are not reported. The lower AUC in HPV-negative patients and the near-perfect AUC values in the base-of-tongue and current-smoker subgroups should be interpreted with caution because these subgroups included very few MLN-negative cases (n = 1–2).

Subgroup	MLN +/−	Sensitivity	Specificity	Balanced Acc	AUC
HPV status: Positive	22/7	0.924	0.579	0.752	0.861
HPV status: Negative	9/2	0.760	0.500	0.630	0.540
HPV status: Unknown	4/6	1.000	1.000	1.000	1.000
PT location: Palatine tonsil	17/5	1.000	0.500	0.750	0.812
PT location: Oral tongue	6/6	0.947	0.882	0.915	0.907
PT location: Base of tongue	9/1	0.828	1.000	0.914	1.000
PT location: Other sites	3/3	0.500	0.833	0.667	0.750
Tobacco use: Non-smoker	16/7	0.857	0.762	0.810	0.900
Tobacco use: Former smoker	15/6	0.902	0.692	0.797	0.812
Tobacco use: Current smoker	4/2	1.000	1.000	1.000	1.000

**Table 6:** Ablation study of cube-based fusion variants for patient-level MLN classification on the test set. Here, *s* denotes the cube size used to reshape the 128-dimensional FLIm feature vector into a 3D cube before volumetric upsampling and fusion with the final MLN-related PET/CT decoder feature map. Cube s2 and Cube s4 correspond to different spatial arrangements of the same FLIm feature vector. Sensitivity, specificity, and balanced accuracy are reported as mean ± standard deviation over 11 random runs, while AUC is computed from pooled subject-level predictions across all runs.

Method	Sensitivity	Specificity	Balanced Acc	AUC
Cube s2	0.878±0.115	0.773±0.221	0.826±0.089	0.842
Cube s4	0.873±0.089	0.780±0.164	0.827±0.093	0.850

**Table 7:** Ablation study of SE-based fusion variants for patient-level MLN classification on the test set. Here, mu and residual denote multiplicative and residual channel-wise modulation, respectively, and *r* denotes the reduction ratio in the SE gating branch. Sensitivity, specificity, and balanced accuracy are reported as mean ± standard deviation over 11 random runs, while AUC is computed from pooled subject-level predictions across all runs.

Method	Sensitivity	Specificity	Balanced Acc	AUC
SE (mul, r=0)	0.888±0.118	0.758±0.195	0.823±0.116	0.833
SE (mul, r=4)	0.857±0.094	0.750±0.211	0.804±0.128	0.814
SE (mul, r=16)	0.899±0.090	0.780±0.221	0.839±0.126	0.872
SE (residual, r=0)	0.876±0.088	0.795±0.141	0.836±0.090	0.851
SE (residual, r=4)	0.875±0.078	0.780±0.221	0.828±0.109	0.845
SE (residual, r=16)	0.895±0.080	0.750±0.247	0.822±0.138	0.828

**Note:** Bold values indicate the best performance for each metric.

**Table 8:** SE gating structures used in the SE-based fusion module for different reduction ratios *r*, along with the corresponding hidden dimensions and parameter counts.

*r*	Structure	Hidden Dim	#Params
0	Linear(128→8)	None	1032
4	Linear(128→32) → ReLU → Linear(32→8)	32	4392
16	Linear(128→8) → ReLU → Linear(8→8)	8	1104

## Data Availability

The data presented in this study are not publicly available due to patient privacy, ethical restrictions, and institutional data-sharing regulations. Data access requests may be directed to the corresponding author and will be considered subject to institutional review board approval and applicable data use agreements. The source code supporting this study is planned to be made publicly available upon publication of the article.

## Supplementary Materials

The following supporting information can be downloaded at: https://www.mdpi.com/article/10.3390/cancers18132154/s1, Figure S1: Absolute Spearman correlation between MLN-branch SE excitation weights and patient-level prediction scores. Channels with higher absolute correlation were more strongly associated with model output; Figure S2: Association between MLN-branch SE excitation weights and FLIm feature groups, summarized by the maximum absolute Spearman correlation within each feature group. These analyses indicate channel-level associations between SE modulation, model prediction, and FLIm-derived feature groups.

## Abbreviations

The following abbreviations are used in this manuscript:

CT	Computed Tomography
FLIm	Fluorescence lifetime imaging
HNC	Head and neck cancer
MLP	Multilayer Perceptron
MLN	Metastatic lymph node
PET	Positron Emission Tomography
PT	Primary tumor
SE	Squeeze-and-excitation
CHUP	Centre Hospitalier Universitaire de Poitiers, France
MDA	MD Anderson Cancer Center, USA
CHUS	Centre Hospitalier Universitaire de Sherbrooke, Canada
HMR	Hôpital Maisonneuve-Rosemont, Canada
HGJ	Hôpital Général Juif/Jewish General Hospital, Canada
CHUM	Centre Hospitalier de l’Université de Montréal, Canada
CHUV	Centre Hospitalier Universitaire Vaudois, Switzerland

## References

[1] Sung H., Ferlay J., Siegel R.L., Laversanne M., Soerjomataram I., Jemal A., Bray F. (2021). Global cancer statistics 2020: GLOBOCAN estimates of incidence and mortality worldwide for 36 cancers in 185 countries. CA A Cancer J. Clin..

[2] Cunha A.R.D., Compton K., Xu R., Mishra R., Drangsholt M.T., Antunes J.L.F., Kerr A.R., Acheson A.R., Lu D., GBD 2019 Lip, Oral, and Pharyngeal Cancer Collaborators (2023). The global, regional, and national burden of adult lip, oral, and pharyngeal cancer in 204 countries and territories: A systematic analysis for the Global Burden of Disease Study 2019. JAMA Oncol..

[3] Xing Y., Zhang J., Lin H., Gold K.A., Sturgis E.M., Garden A.S., Lee J.J., William W.N. (2016). Relation between the level of lymph node metastasis and survival in locally advanced head and neck squamous cell carcinoma. Cancer.

[4] Valizadeh P., Jannatdoust P., Pahlevan-Fallahy M.T., Hassankhani A., Amoukhteh M., Bagherieh S., Ghadimi D.J., Gholamrezanezhad A. (2025). Diagnostic accuracy of radiomics and artificial intelligence models in diagnosing lymph node metastasis in head and neck cancers: A systematic review and meta-analysis. Neuroradiology.

[5] D’Cruz A.K., Vaish R., Kapre N., Dandekar M., Gupta S., Hawaldar R., Agarwal J.P., Pantvaidya G., Chaukar D., Deshmukh A. (2015). Elective versus therapeutic neck dissection in node-negative oral cancer. N. Engl. J. Med..

[6] Hoang J.K., Vanka J., Ludwig B.J., Glastonbury C.M. (2013). Evaluation of cervical lymph nodes in head and neck cancer with CT and MRI: Tips, traps, and a systematic approach. Am. J. Roentgenol..

[7] Morisada M.V., Bewley A.F., Broadhead K., Assadsangabi R., Paydar A., Birkeland A.C., Abouyared M., Qi L., Ivanovic V. (2024). CT predictors of sub-centimeter occult lymph node metastases in oral cavity squamous cell carcinoma: A case-control study. Neuroradiol. J..

[8] Jović A., Fila J., Gršić K., Ivkić M., Ozretić D. (2020). Diffusion-weighted MRI: Impact of the size of the ROI in detecting metastases in subcentimeter lymph nodes in head and neck squamous cell carcinoma. Neuroradiology.

[9] Psychogios G., Mantsopoulos K., Bohr C., Koch M., Zenk J., Iro H. (2013). Incidence of occult cervical metastasis in head and neck carcinomas: Development over time. J. Surg. Oncol..

[10] Tomita H., Yamashiro T., Heianna J., Nakasone T., Kimura Y., Mimura H., Murayama S. (2021). Nodal-based radiomics analysis for identifying cervical lymph node metastasis at levels I and II in patients with oral squamous cell carcinoma using contrast-enhanced computed tomography. Eur. Radiol..

[11] Xu X., Xi L., Wei L., Wu L., Xu Y., Liu B., Li B., Liu K., Hou G., Lin H. (2023). Deep learning assisted contrast-enhanced CT–based diagnosis of cervical lymph node metastasis of oral cancer: A retrospective study of 1466 cases. Eur. Radiol..

[12] Yuan Y., Ren J., Tao X. (2021). Machine learning–based MRI texture analysis to predict occult lymph node metastasis in early-stage oral tongue squamous cell carcinoma. Eur. Radiol..

[13] Lan T., Kuang S., Liang P., Ning C., Li Q., Wang L., Wang Y., Lin Z., Hu H., Yang L. (2024). MRI-based deep learning and radiomics for prediction of occult cervical lymph node metastasis and prognosis in early-stage oral and oropharyngeal squamous cell carcinoma: A diagnostic study. Int. J. Surg..

[14] Konishi M., Kakimoto N. (2023). Radiomics analysis of intraoral ultrasound images for prediction of late cervical lymph node metastasis in patients with tongue cancer. Head. Neck.

[15] Fukuda M., Eida S., Katayama I., Takagi Y., Sasaki M., Sumi M., Ariji Y. (2025). A radiomics model combining machine learning and neural networks for high-accuracy prediction of cervical lymph node metastasis on ultrasound of head and neck squamous cell carcinoma. Oral Surg. Oral Med. Oral Pathol. Oral Radiol..

[16] Alsibani A., Alqahtani A., Almohammadi R., Islam T., Alessa M., Aldhahri S.F., Al-Qahtani K.H. (2024). Comparing the efficacy of CT, MRI, PET-CT, and US in the detection of cervical lymph node metastases in head and neck squamous cell carcinoma with clinically negative neck lymph node: A systematic review and meta-analysis. J. Clin. Med..

[17] Chen L., Dohopolski M., Zhou Z., Wang K., Wang R., Sher D., Wang J. (2021). Attention guided lymph node malignancy prediction in head and neck cancer. Int. J. Radiat. Oncol. Biol. Phys..

[18] Dohopolski M., Chen L., Sher D., Wang J. (2020). Predicting lymph node metastasis in patients with oropharyngeal cancer by using a convolutional neural network with associated epistemic and aleatoric uncertainty. Phys. Med. Biol..

[19] Chen L., Zhou Z., Sher D., Zhang Q., Shah J., Pham N.L., Jiang S., Wang J. (2019). Combining many-objective radiomics and 3D convolutional neural network through evidential reasoning to predict lymph node metastasis in head and neck cancer. Phys. Med. Biol..

[20] Marsden M., Weyers B.W., Bec J., Sun T., Gandour-Edwards R.F., Birkeland A.C., Abouyared M., Bewley A.F., Farwell D.G., Marcu L. (2020). Intraoperative margin assessment in oral and oropharyngeal cancer using label-free fluorescence lifetime imaging and machine learning. IEEE Trans. Biomed. Eng..

[21] Hassan M.A., Weyers B.W., Bec J., Fereidouni F., Qi J., Gui D., Bewley A.F., Abouyared M., Farwell D.G., Birkeland A.C. (2023). Anatomy-specific classification model using label-free FLIm to aid intraoperative surgical guidance of head and neck cancer. IEEE Trans. Biomed. Eng..

[22] Johnson D.E., Burtness B., Leemans C.R., Lui V.W.Y., Bauman J.E., Grandis J.R. (2020). Head and neck squamous cell carcinoma. Nat. Rev. Dis. Prim..

[23] Yuan N., Hassan M.A., Ehrlich K., Weyers B.W., Biddle G., Ivanovic V., Raslan O.A., Gui D., Abouyared M., Bewley A.F. (2024). Early detection of lymph node metastasis using primary head and neck cancer computed tomography and fluorescence lifetime imaging. Diagnostics.

[24] Gorpas D., Phipps J., Bec J., Ma D., Dochow S., Yankelevich D., Sorger J., Popp J., Bewley A., Gandour-Edwards R. (2019). Autofluorescence lifetime augmented reality as a means for real-time robotic surgery guidance in human patients. Sci. Rep..

[25] Marsden M., Fukazawa T., Deng Y.C., Weyers B.W., Bec J., Gregory Farwell D., Marcu L. (2020). FLImBrush: Dynamic visualization of intraoperative free-hand fiber-based fluorescence lifetime imaging. Biomed. Opt. Express.

[26] Liu J., Sun Y., Qi J., Marcu L. (2012). A novel method for fast and robust estimation of fluorescence decay dynamics using constrained least-squares deconvolution with Laguerre expansion. Phys. Med. Biol..

[27] Zhou L., Yuan N., Ehrlich K., Qi J. (2025). NCF: Neural correspondence field for medical image registration. Proceedings of the Medical Imaging 2025: Image Processing.

[28] Meng M., Bi L., Fulham M., Feng D., Kim J. (2023). Merging-diverging hybrid transformer networks for survival prediction in head and neck cancer. Proceedings of the International Conference on Medical Image Computing and Computer-Assisted Intervention.

[29] Andrearczyk V., Oreiller V., Boughdad S., Le Rest C.C., Tankyevych O., Elhalawani H., Jreige M., Prior J.O., Vallières M., Visvikis D. (2023). Automatic head and neck tumor segmentation and outcome prediction relying on FDG-PET/CT images: Findings from the second edition of the HECKTOR challenge. Med. Image Anal..

[30] Milletari F., Navab N., Ahmadi S.A. (2016). V-net: Fully convolutional neural networks for volumetric medical image segmentation. Proceedings of the 2016 Fourth International Conference on 3D Vision (3DV).

[31] Lin T.Y., Goyal P., Girshick R., He K., Dollár P. Focal loss for dense object detection. Proceedings of the IEEE International Conference on Computer Vision.

[32] Hu J., Shen L., Sun G. Squeeze-and-excitation networks. Proceedings of the IEEE Conference on Computer Vision and Pattern Recognition.

[33] Meeks N., McAdoo A.G., Lee Y.J., Akhund R., Smith G., Grice J., Hoang J., Zinn K.R., Hom M.E., Topf M.C. (2026). Use of Dual-Modality Antibody Imaging for Assessment of Lymph Node Metastases in Head and Neck Cancer. Theranostics.

