# Supplementary material for: Multimodal Fusion of Intraoperative FLIm and Preoperative PET/CT for Patient-Level Prediction of Lymph Node Metastasis in Head and Neck Cancer
Source: Cancers (Basel). 2026 Jul 4;18(13):2154. doi: 10.3390/cancers18132154 (PMC13359770; doi:10.3390/cancers18132154)
Supplement: Supplementary file 1 [file cancers-18-02154-s001.zip › cancers-4371483-supplementary.pdf]

Supplementary Materials: Multimodal Fusion of Intraoperative FLIm and Preoperative PET/CT for Patient-Level Prediction of Lymph Node Metastasis in Head and Neck Cancer

Lei Zhou [1], Nimu Yuan [1], Mohamed A. Hassan [1], Lisanne Kraft [1], Katjana Ehrlich [1], Brent W. Weyers [1], Vladimir Ivanovic [2,3], Osama A. A. Raslan [3], Dorina Gui [4], Marianne Abouyared [5], Arnaud F. Bewley [5], Andrew C. Birkeland [5], Donald Gregory Farwell [6], Laura Marcu [1,7] and Jinyi Qi [1,*]

Supplementary Model Interpretation Analysis

To examine the behavior of the SE fusion module, we analyzed patient-level excitation weights from the MLN branch. The absolute Spearman correlation between each channel weight and the corresponding prediction score was used to identify channels more strongly associated with model output (Figure S1). For each channel, associations with clinical and FLIm feature groups were summarized using the maximum absolute Spearman correlation among the variables within each group. As shown in Figure S2, prediction-associated SE channels were related to multiple feature groups, including intensity ratio, Laguerre coefficients, fluorescence lifetime, spectral intensity, and clinical variables. These exploratory results suggest that SE Fusion integrates multiple FLIm-derived optical-biochemical feature groups through channel-wise modulation of PET/CT representations.

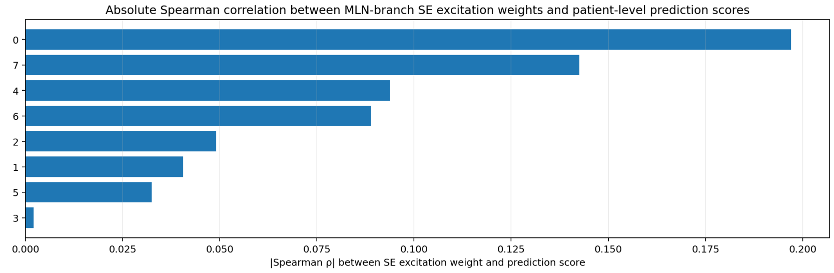

Figure S1. Absolute Spearman correlation between MLN-branch SE excitation weights and patient-level prediction scores. Channels with higher absolute correlation were more strongly associated with model output.

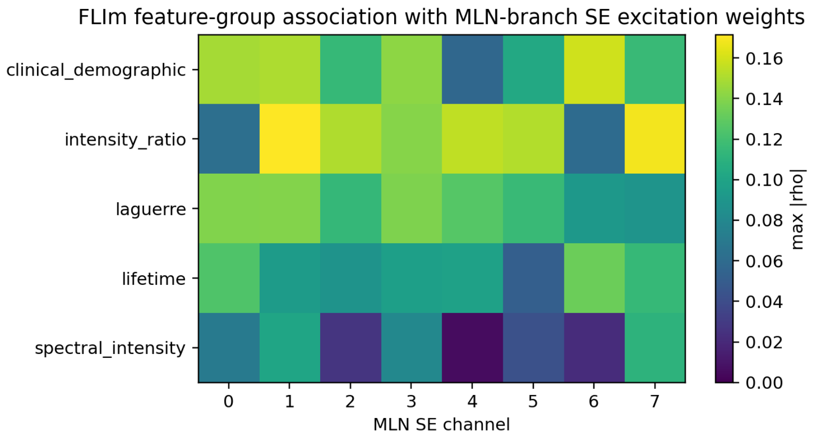

Figure S2. Association between MLN-branch SE excitation weights and FLIm feature groups, summarized by the maximum absolute Spearman correlation within each feature group. These analyses indicate channel-level associations between SE modulation, model prediction, and FLIm-derived feature groups.
